# Mitochondria-Targeted Antioxidant Prevents Cardiac Dysfunction Induced by Tafazzin Gene Knockdown in Cardiac Myocytes

**DOI:** 10.1155/2014/654198

**Published:** 2014-08-27

**Authors:** Quan He, Nicole Harris, Jun Ren, Xianlin Han

**Affiliations:** ^1^Diabetes and Obesity Research Center, Sanford-Burnham Medical Research Institute, 6400 Sanger Road, Orlando, FL 32827, USA; ^2^Center for Cardiovascular Research and Alternative Medicine, University of Wyoming, Laramie, WY 82071, USA

## Abstract

Tafazzin, a mitochondrial acyltransferase, plays an important role in cardiolipin side chain remodeling. Previous studies have shown that dysfunction of tafazzin reduces cardiolipin content, impairs mitochondrial function, and causes dilated cardiomyopathy in Barth syndrome. Reactive oxygen species (ROS) have been implicated in the development of cardiomyopathy and are also the obligated byproducts of mitochondria. We hypothesized that tafazzin knockdown increases ROS production from mitochondria, and a mitochondria-targeted antioxidant prevents tafazzin knockdown induced mitochondrial and cardiac dysfunction. We employed cardiac myocytes transduced with an adenovirus containing tafazzin shRNA as a model to investigate the effects of the mitochondrial antioxidant, mito-Tempo. Knocking down tafazzin decreased steady state levels of cardiolipin and increased mitochondrial ROS. Treatment of cardiac myocytes with mito-Tempo normalized tafazzin knockdown enhanced mitochondrial ROS production and cellular ATP decline. Mito-Tempo also significantly abrogated tafazzin knockdown induced cardiac hypertrophy, contractile dysfunction, and cell death. We conclude that mitochondria-targeted antioxidant prevents cardiac dysfunction induced by tafazzin gene knockdown in cardiac myocytes and suggest mito-Tempo as a potential therapeutic for Barth syndrome and other dilated cardiomyopathies resulting from mitochondrial oxidative stress.

## 1. Introduction

Tafazzin is a mitochondrial phospholipid-lysophospholipid acyltransferase [1, 2]. It plays important role in cardiolipin side chains remodeling from their nascent forms to tetralinoleoyl cardiolipin, which is believed to be the functional species in adult mammal heart [3]. Cardiolipin, a mitochondrial signature phospholipid consisting of two phosphatidylglycerols, is essential for optimal mitochondrial function. Cardiolipin is initially synthesized as a premature form and becomes fully functional when its four fatty acid chains are remodeled by enzymes including tafazzin [1, 4]. Tafazzin is encoded by the* G4.5* gene in humans [5]. Tafazzin mutation causes Barth syndrome, a rare and often fatal x-linked genetic disorder which is characterized by aciduria, neutropenia, dilated cardiomyopathy, and myocardial noncompaction [6, 7]. Heart failure and arrhythmias are the causes of death in the early childhood. Dysfunction of the tafazzin gene reduces cardiolipin and impairs mitochondrial structure and function in yeast and in patients with Barth syndrome [8–11]. Most of the cardiac abnormalities of Barth syndrome have been mimicked in animal models, for example, reduced locomotor activity in* Drosophila* [12], signs of Barth syndrome heart failure in zebrafish [13], and cardiomyopathies in mice [14, 15]. Decreased cardiolipin contents and impaired mitochondrial function due to tafazzin dysfunction have been demonstrated in several cell models including yeast [16], human lymphoblasts [11], neonatal cardiac fibroblasts, and cardiac myocytes [17, 18]. Tafazzin knockdown resulted in cardiac hypertrophy in neonatal cardiac myocytes [17]. Impaired mitochondrial function is the potential cause of cardiomyopathy seen in Barth syndrome since cardiomyopathy is also a common clinical presentation of mitochondrial disease [19–21].

Our previous study showed that tafazzin knockdown enhances mitochondrial reactive oxygen species (ROS) production in neonatal cardiac fibroblasts [18]. Tafazzin mutation causes oxidative stress in yeast as well [22]. Imbalanced ROS have been implicated in the pathogenesis of a variety of diseases, including diabetes [23], neurodegenerative diseases [24], ischemia-reperfusion injury [25], and heart failure [26]. Mitochondria occupy about 30% of the cardiac myocyte volume and are postulated to be the major cellular ROS source. ROS are the byproducts of the mitochondrial respiration chain complexes. Up to 2% of the oxygen consumed in the respiration chain was used to form superoxide in a quiescent condition [27]. ROS are also produced from mitochondrial enzymatic reactions catalyzed by aconitase, α-ketoglutarate dehydrogenase, pyruvate dehydrogenase, glycerol-3-phosphate dehydrogenase, dihydroorotate dehydrogenase, monoamine oxidase, and cytochrome b5 reductase [28]. Antioxidation defense systems exist in the mitochondria, including manganese superoxide dismutase (Mn-SOD), catalase, glutathione peroxidase, and thioredoxin peroxidase [28, 29]. Besides mitochondria, cellular ROS are also contributed by xanthine oxidase, NADPH oxidase, and uncoupled nitric oxide synthase [30]. The importance of mitochondrial ROS has demonstrated that mice null for Mn-SOD exhibit lethality due to cardiac dysfunction [31], heart/muscle-specific ablation of Mn-SOD produces progressive congestive heart failure [32], and overexpression of a mitochondrial ROS scavenger, peroxiredoxin3, prevents heart failure induced by myocardial infarction [33]. Clinical investigation showed that general antioxidant supplement has no beneficial effects on cardiovascular diseases [34]; however, the mitochondria-targeted antioxidant MitoQ10 proved effective for endothelial improvement and cardiac hypertrophy attenuation in stroke-prone spontaneously hypertensive rats [35].

Our current study was designed to demonstrate that mitochondrial ROS play critical roles in tafazzin knockdown induced cardiac and mitochondrial dysfunction in cultured cardiac myocytes. We found that tafazzin knockdown enhanced ROS production from the mitochondria, and a mitochondria-targeted antioxidant normalized tafazzin knockdown induced ATP decline, cardiac myocyte hypertrophy, contractile dysfunction, and cell death.

## 2. Materials and Methods

Animal protocols under IACUC #2011-0059 were approved by the Institutional Animal Care and Use Committee of the Sanford-Burnham Medical Research Institute, Orlando Diabetes and Obesity Research Center, Florida.

### 2.1. Supplies and Chemicals

Phosphatase and proteinase inhibitor cocktail tablets (PhosSTOP and Complete Mini) were obtained from Roche Applied Science (Indianapolis, IN). Primary antibodies against phospho-AMPKα (Thr172), phospho-Jak2 (Tyr107/108), cytochrome c, and *β*-actin and a horseradish peroxidase-conjugated secondary antibody against rabbit IgG were purchased from Cell Signaling Technology (Boston, MA). The antibody against tafazzin was from Santa Cruz (Santa Cruz, CA). Coomassie protein assay kit, MemCode reversible protein stain kit, mitochondria isolation kit for mammalian cells, SuperSignal West Pico chemiluminescent substrates, and Restore Plus Western blot stripping buffer were purchased from Thermo Scientific (Rockford, IL). Cell culture medium and supplements, precast tris-glycine polyacrylamide gels, polyvinylidene fluoride (PVDF) membranes, SYBR Green PCR Master Mix, MitoTracker Green, and the mitochondrial superoxide indicator MitoSOX Red were obtained from Life Technologies (San Diego, CA). ^3^H-leucine was purchased from PerkinElmer (Waltham, MA). Random primers and Omniscript reverse transcriptase were obtained from Qiagen (Valencia, CA). Custom primers were synthesized by TIB MolBiol (Adelphia, NJ). The tafazzin short hairpin RNA (shRNA) adenovirus (pSilencer adeno 1.0-CMV) was expanded and purified by the Gene Therapy Center Virus Vector Core Facility of the University of North Carolina at Chapel Hill. An ATP assay kit was purchased from BioVision (Milpitas, CA). The Tunel apoptosis detection kit was from EMD Millipore (Billerica, MA). Hematoxylin and eosin reagents were obtained from VWR (Atlanta, GA). Mito-Tempo and Tempol were obtained from ENZO Life Sciences (Farmingdale, NY). Other routine supplies and chemicals were purchased from Fisher and Sigma. A muscle derived cell line C2C12 was obtained from ATCC (Manassas, VA).

### 2.2. Cell Culture

Neonatal ventricular myocytes (NVMs) were generated from 1-day-old Sprague-Dawley rat pups (Charles River Laboratories) as described previously [17]. Cells were cultured in DMEM containing 100 U/mL penicillin, 100 *μ*g/mL streptomycin, 2 mM glutamine, and 10% fetal bovine serum in a humidified CO_2_ incubator at 37°C. Cells were treated in glucose-free DMEM after being serum-starved for 24 h.

Adult cardiac myocytes (ACMs) were isolated from 9-week old C57/B6 male mice as described previously [36] by retrograde perfusion of digestion buffer containing liberase (0.25 mg/mL) and trypsin (0.14 mg/mL). The cells were preplated in a MEM medium supplement with 5% fetal bovine serum and 10 mM 2,3-butanedione monoxime (BDM) to stop cell contraction for 1 h and then cultured in MEM containing 0.1 mg/mL bovine serum albumin, 10 mM BDM, insulin-transferrin-selenium, and antibiotics.

Adenoviral transduction of cardiac myocytes, protein extraction and Western blot, ^3^H-leucine incorporation, and ATP assay were performed as described previously [17].

### 2.3. Cardiolipin Analysis

Cells were scraped into PBS and the pellets kept at −80°C until just prior to processing. Lipids were extracted and cardiolipin analyzed by shotgun lipidomics [37].

### 2.4. Mitochondrial Reactive Oxygen Species (ROS)

Cells were incubated with 300 nM MitoTracker Green in serum-free medium at 37°C for 45 min. The cells were washed with HBSS (Hank's balanced salt solution) and incubated with 5 *μ*M MitoSOX red at 37°C for 5 min in HBSS. The cells were kept in HBSS after washing. Five images were captured from each field using fluorescence microscopy with green and red filters and merged with Elements software. The red fluorescence intensity representing mitochondrial ROS was quantified using Image J and expressed as the corrected total cell fluorescence.

### 2.5. Mitochondrial Cytochrome C Assay

Mitochondria were isolated from NVMs with a kit from Thermo Fisher following the manufacturer's directions. The isolated mitochondria were dissolved in protein sample buffer by boiling. The mitochondrial protein samples were separated on 16% SDS polyacrylamide gel electrophoresis and transferred onto PVDF membranes. The major protein bands were captured for loading control after visualization using a MemCode reversible protein stain kit. After destaining, the PVDF membrane was subjected to Western blot for cytochrome c. The band intensity was quantified, corrected for the major protein bands, and expressed as a percentage of scrambled virus treated with vehicle.

### 2.6. Real-Time RT-PCR

Total RNA isolation from NVMs and real-time RT-PCR with the primers for tafazzin and *β*-actin in an Eppendorf RealPlex2 were performed as we described previously [17]. Target gene mRNA levels were determined using the ΔΔCt method [38] and expressed as relative to the control, which was NVMs infected with a scrambled adenovirus.

### 2.7. Cell Surface Area

NVMs were plated onto laminin-coated coverslips in 6-well plates at a density of 0.25 million cells per well. After treatments, cells were fixed with 3.7% formaldehyde for 20 min at room temperature. The fixed cells were stained with hematoxylin/eosin and mounted. Five images of each sample were acquired under an Olympus I × 71 inverted microscope. The cell surface area was analyzed with Image J.

### 2.8. Cell Count

Cells were trypsinized and fixed with 3.7% formaldehyde in PBS for 20 min at room temperature. Cells were counted with a hemocytometer.

### 2.9. Myocyte Contractility

ACMs were plated on laminin coated coverslips, incubated with tafazzin shRNA adenovirus for 24 h and treated with mito-Tempo overnight. They were stimulated at 1 Hz with a MyoPacer electric field in MEM culture medium containing 1.8 mM calcium at room temperature. Sarcomere length was measured with an IonWizars system from IonOptix. Myocyte contractility was represented by sarcomere shortening, for example, percent change in length.

### 2.10. Statistical Analysis

Data were expressed as mean ± SE and differences in mean values were analyzed using an unpaired 2-tailed *t*-test. *P* < 0.05 was considered statistically significant.

## 3. Results

### 3.1. Tafazzin Knockdown Decreased Cardiolipin

**Figure 1:**
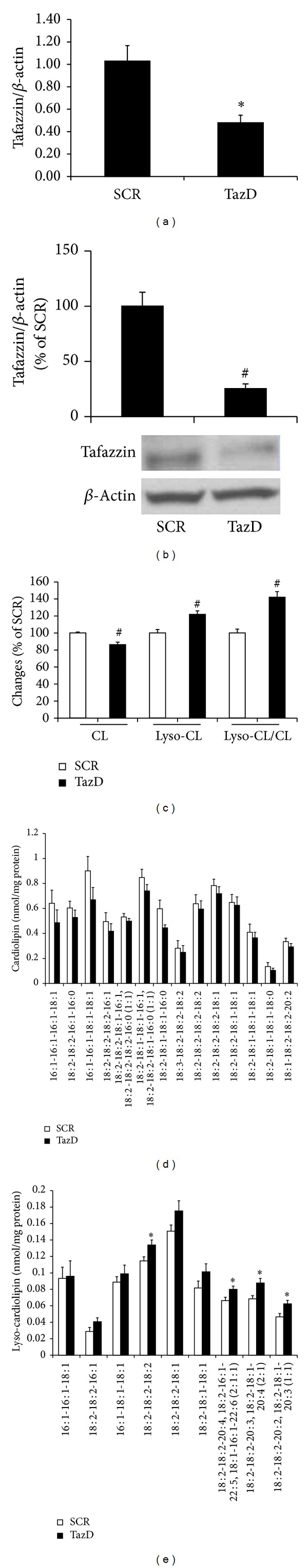
Tafazzin shRNA adenovirus knocked down tafazzin expression and cardiolipin. NVMs were transduced with the tafazzin (TazD) shRNA adenovirus for 48 h and tested for tafazzin expression ((a) and (b)) and cardiolipin ((c), (d), and (e)). (a) Tafazzin mRNA was measured by real-time RT-PCR using *β*-actin as a normalizer and expressed as relative level compared to the control (scrambled adenovirus or SCR). Data represent mean ± SE of 4 separate experiments. *P < 0.05 versus SCR. (b) Tafazzin protein was determined by Western blot and expressed as a percentage of SCR. Data represent mean ± SE of 3 separate experiments. ^#^ *P* < 0.01 versus SCR. (c) Cardiolipin (CL) and monolysocardiolipin (lyso-CL) were identified and quantified by mass spectrometry. Data represent mean ± SE of 9 separate experiments. ^#^ *P* < 0.01 versus SCR. The basal contents (including all detected species) of cardiolipin and monolysocardiolipin were 8.07 ± 0.36 and 0.92 ± 0.04 nmol/mg protein, respectively. Species of cardiolipin (d) and monolysocardiolipin (e) were identified and quantified by shotgun lipidomics. Data represent mean ± SE of 8 separate experiments. *P < 0.05 versus SCR.

To see if tafazzin shRNA would knock down tafazzin expression, NVMs were transduced with the tafazzin shRNA adenovirus for 48 h and its expression measured by real-time RT-PCR and Western blot. We found that the tafazzin shRNA adenovirus significantly knocked down both tafazzin mRNA and protein compared with the scrambled adenovirus (Figures 1(a) and 1(b)). Since tafazzin plays an important role in cardiolipin remodeling, and since mutation of tafazzin decreases cardiolipin, we measured cardiolipin by shotgun lipidomics and found that tafazzin knockdown reduced cardiolipin to 86% while increasing both monolysocardiolipin (to 122%) and the ratio of monolysocardiolipin to cardiolipin (to 142%) compared control NVMs treated with scrambled virus (Figure 1(c)). Side chain analysis showed that cardiolipin species were deeply redistributed and shifted to the short chain. While most cardiolipin species tended to decrease (Figure 1(d)), monolysocardiolipin tended to increase (Figure 1(e)). Since there is no predominant species, we suggested that total cardiolipin plays more important roles than specific cardiolipin species in neonatal cardiac myocyte.

### 3.2. Tafazzin Knockdown Enhanced Mitochondrial ROS Production, which Is Abrogated by the Mitochondrial Antioxidant

**Figure 2:**
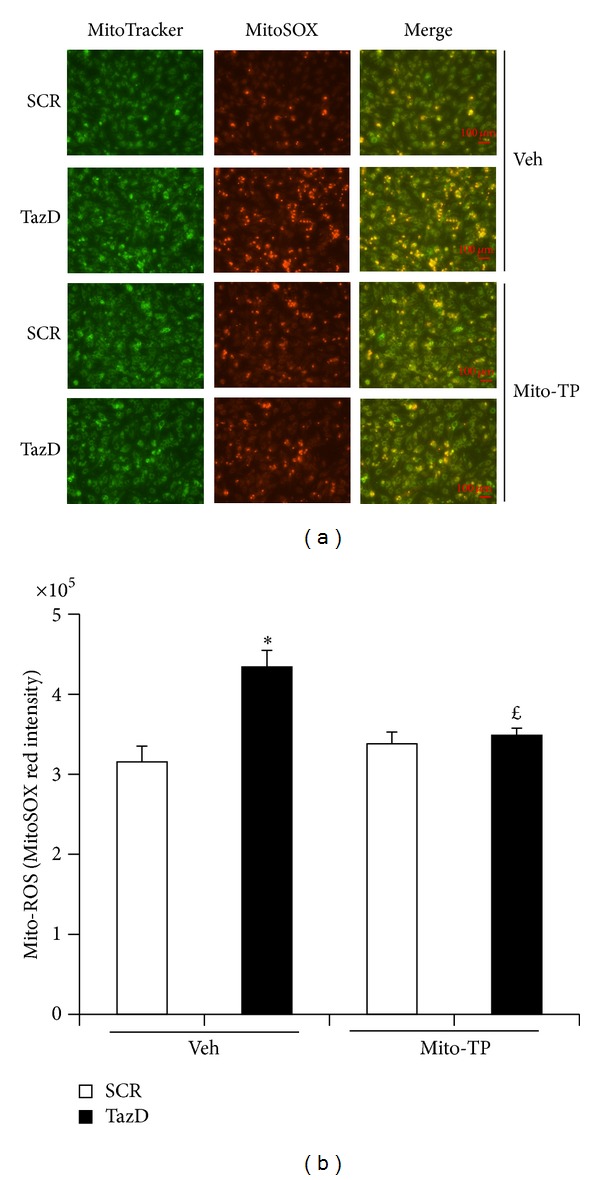
Mito-Tempo blocked the enhanced mitochondrial ROS production induced by tafazzin knockdown. NVMs were transduced with the shRNA adenovirus overnight, treated with mito-Tempo for 24 h and tested for ROS. (a) Representative picture of mitochondrial ROS. Each panel represents 15 images from 3 separate experiments. Scale bars, 100 *μ*m. (b) Quantitative analysis of panel A MitoSOX red staining. Data represent mean ± SE of 3 separate experiments. *P < 0.05 versus SCR treated with vehicle. ^*£*^ *P* < 0.05 versus Taz knockdown cells treated with vehicle.

Cardiolipin plays an important role in maintaining optimal function of the mitochondrial respiration chain, and dysfunction of mitochondria increases ROS production. We found that tafazzin knockdown obviously increased mitochondrial ROS production as assayed by MitoSOX red staining. The enhanced MitoSOX red stain was abolished by mito-Tempo, a mitochondria-specific antioxidant, which is antioxidant tempol covalently attached to lipophilic triphenylphosphonium cation. The alkyltriphenylphosphonium cations are preferably accumulated in mitochondria by mitochondrial membrane potential [39]. The MitoSOX red stain overlapped perfectly with MitoTracker green (Figure 2(a)). After quantification analysis, we found that the enhanced mitochondrial ROS production was blocked by the treatment with 25 *μ*M mito-Tempo (Figure 2(b)).

### 3.3. Mitochondrial Antioxidant Normalized ATP Decline Induced by Tafazzin Knockdown

**Figure 3:**
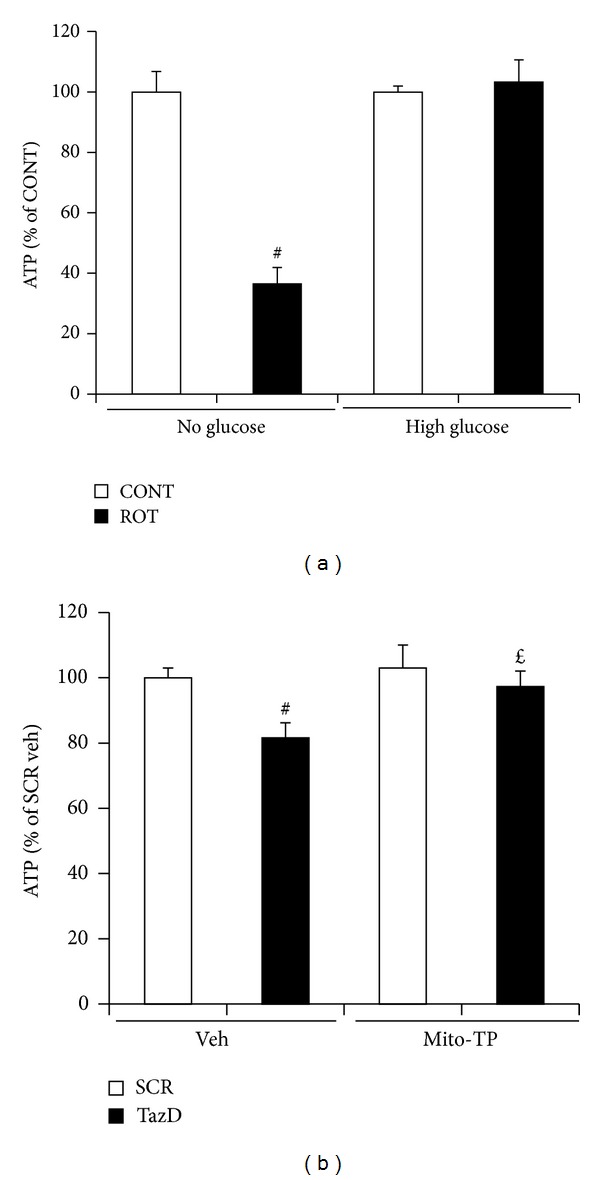
Mito-Tempo abolished ATP shortage induced by tafazzin knockdown. (a) C2C12 cells were cultured in high glucose DMEM to 70% confluence, serum starved, and treated with 5 nM rotenone (ROT) in glucose-free or high glucose DMED for 24 h. Data represent mean ± SE of 3 separate experiments. ^#^ *P* < 0.05 versus control cells which were treated with DMSO. (b). NVMs were transduced with the shRNA adenovirus for 48 h, treated with mito-Tempo in the last 24 h, and tested for ATP with a kit from BioVision. Data represent mean ± SE of 4 separate experiments. ^#^ *P* < 0.01 versus SCR treated with vehicle. ^*£*^ *P* < 0.05 versus Taz knockdown cells treated with vehicle.

Our previous study showed that tafazzin knockdown decreases ATP production from the mitochondria [17], we next tested whether mito-Tempo affects intracellular ATP. The intracellular ATP was mainly from mitochondrial oxidative phosphorylation, since we used glucose-free DMEM. The only fuel molecule in our culture system is glutamine, which is used to produce ATP in the mitochondria. As showed in Figure 3(a), inhibition of mitochondrial respiration chain complex I with 5 nM rotenone dramatically decreased cellular ATP in C2C12 cells cultured in glucose-free medium but had no effect if the cells were cultured in glucose containing medium. Our results also showed that mito-Tempo normalized ATP decline induced by tafazzin knockdown compared with the vehicle treated cells in the glucose-free medium (Figure 3(b)).

### 3.4. Mitochondrial Antioxidant Prevented Protein Kinases Activation Induced by Tafazzin Knockdown

**Figure 4:**
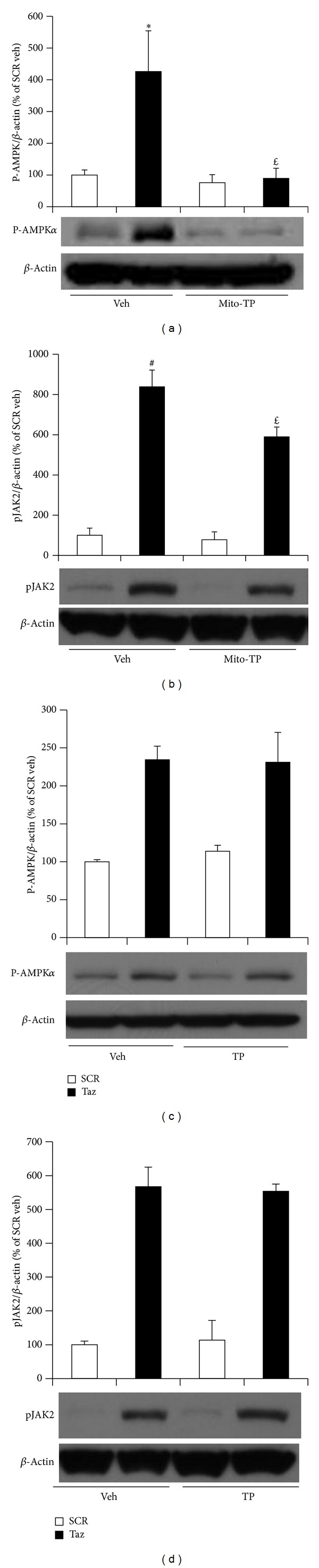
Mito-Tempo inhibited tafazzin knockdown induced protein kinases. NVMs were transduced with the shRNA adenovirus for 48 h, treated with 25 *μ*M mito-Tempo (mito-TP) or Tempol (TP) for the last 24 h, and tested for phosphorylated AMPKα ((a) and (c)) and JAK2 ((b) and (d)). Data represent mean ± SE of 5 or 3 separate experiments for p-AMPKα with the treatments of mito-TP or TP and 4 separate experiments for JAK2 with the treatments mito-TP or TP. *P < 0.05 and ^#^ *P* < 0.01 versus SCR treated with vehicle. ^*£*^ *P* < 0.05 versus Taz knockdown cells treated with vehicle.

Our previous study showed that tafazzin knockdown activates AMPK and is involved in mitochondrial biogenesis, which contributes to cardiac myocyte hypertrophy [17]. As shown in Figure 4, AMPK was significantly activated (*via* increases in its phosphorylation) by tafazzin knockdown and this activation was blocked by mito-Tempo (Figure 4(a)). ROS reportedly activate Janus kinase (JAK) involved in cardiac hypertrophy [40, 41]. We tested whether tafazzin knockdown activates JAK and the effects of mito-Tempo on JAK activation. We found that tafazzin knockdown dramatically activated JAK2 by increasing its phosphorylation. Mito-Tempo significantly abrogated tafazzin knockdown induced JAK2 phosphorylation (Figure 4(b)). A cell permeable antioxidant Tempol (25 *μ*M) was failed to inhibit tafazzin knockdown induced phosphorylation of AMPK or JAK2 (Figures 4(c) and 4(d)).

### 3.5. Mitochondrial Antioxidant Prevented Cardiac Myocyte Hypertrophy Induced by Tafazzin Knockdown

**Figure 5:**
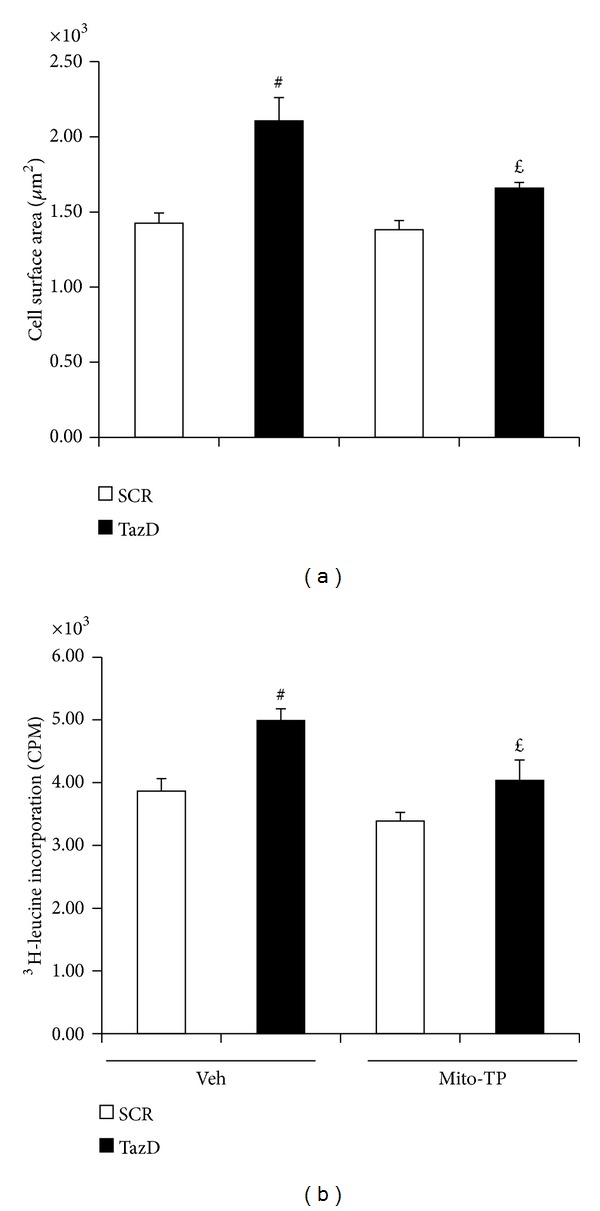
Mito-Tempo prevented hypertrophy induced by tafazzin knockdown. NVMs were transduced with the shRNA adenovirus for 48 h and assayed for hypertrophy. (a) Cell surface area. NVMs were fixed and stained for cell size. Data represent mean ± SE of 4 separate experiments. ^#^ *P* < 0.01 versus SCR treated with vehicle and ^*£*^ *P* < 0.05 versus TazD treated with vehicle. (b). ^3^H-leucine incorporation. Data represent mean ± SE of 4 separate experiments. ^#^ *P* < 0.01 versus SCR treated with vehicle and ^*£*^ *P* < 0.05 versus TazD treated with vehicle.

Our previous study showed that tafazzin knockdown induces cardiac myocyte hypertrophy [17]. We questioned whether mito-Tempo affects hypertrophy. As showed in Figure 5(a), tafazzin knockdown increased cell surface area which is a marker of hypertrophy. This effect of tafazzin knockdown on cell surface area was blocked by the treatment of mito-Tempo. Further analysis revealed that mito-Tempo significantly attenuated not only cardiac myocyte surface area but also protein synthesis as assayed by ^3^H-leucine incorporation (Figure 5(b)). We conclude that mito-Tempo blocked cardiac hypertrophy induced by tafazzin knockdown.

### 3.6. Mitochondrial Antioxidant Prevented Cardiac Myocyte Contractile Dysfunction Induced by Tafazzin Knockdown

**Figure 6:**
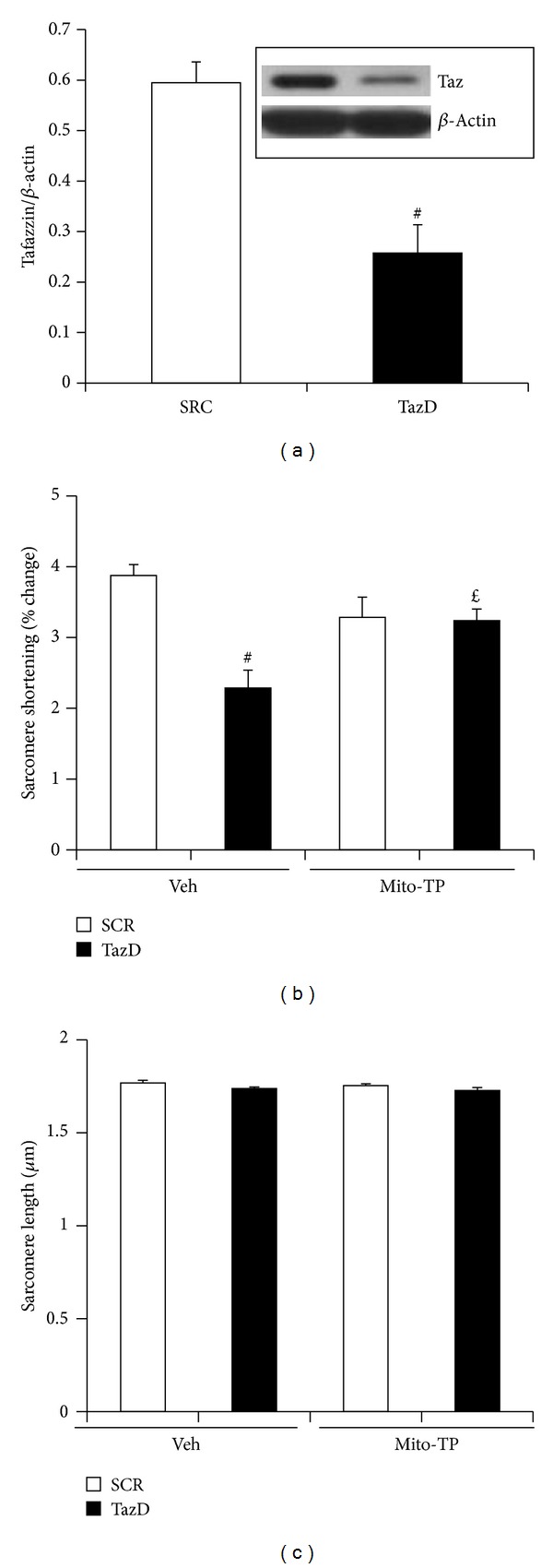
Mito-Tempo normalized contractile dysfunction induced by cardiolipin deficiency due to tafazzin knockdown. Cardiac myocytes isolated from adult mouse hearts were transduced with tafazzin shRNA adenovirus and treated with mito-Tempo overnight. (a) Tafazzin protein. Adenovirus containing tafazzin shRNA efficiently knocked down tafazzin protein. Data represent mean ± SE of 3 separate experiments. ^#^ *P* < 0.01 versus SCR. (b) Sarcomere shortening. Data represent mean ± SE of 4 separate experiments. ^#^ *P* < 0.01 versus SCR treated with vehicle and ^*£*^ *P* < 0.05 versus TazD treated with vehicle. (c) Sarcomere length. Data represent mean ± SE of 4 separate experiments.

Tafazzin mutation causes impaired cardiac function in Barth syndrome patients [42] and tafazzin knockdown in mice reduces cardiac and skeletal muscle contractility [14, 43]. We questioned whether tafazzin knockdown impairs cardiac myocyte contractility and the effects of mito-Tempo treatment. As shown in Figure 6(a), tafazzin shRNA adenovirus significantly knocked down tafazzin protein in ACM. Tafazzin knockdown decreased cardiac myocyte contractility as measured by sarcomere shortening. The treatment of Mito-Tempo prevented tafazzin knockdown- induced contractile dysfunction (Figure 6(b)). Tafazzin knockdown did not affect sarcomere basal length at relaxation (Figure 6(c)).

### 3.7. Mitochondrial Antioxidant Prevents Cytochrome C Release from the Mitochondria and Cell Death Induced by Tafazzin Knockdown

**Figure 7:**
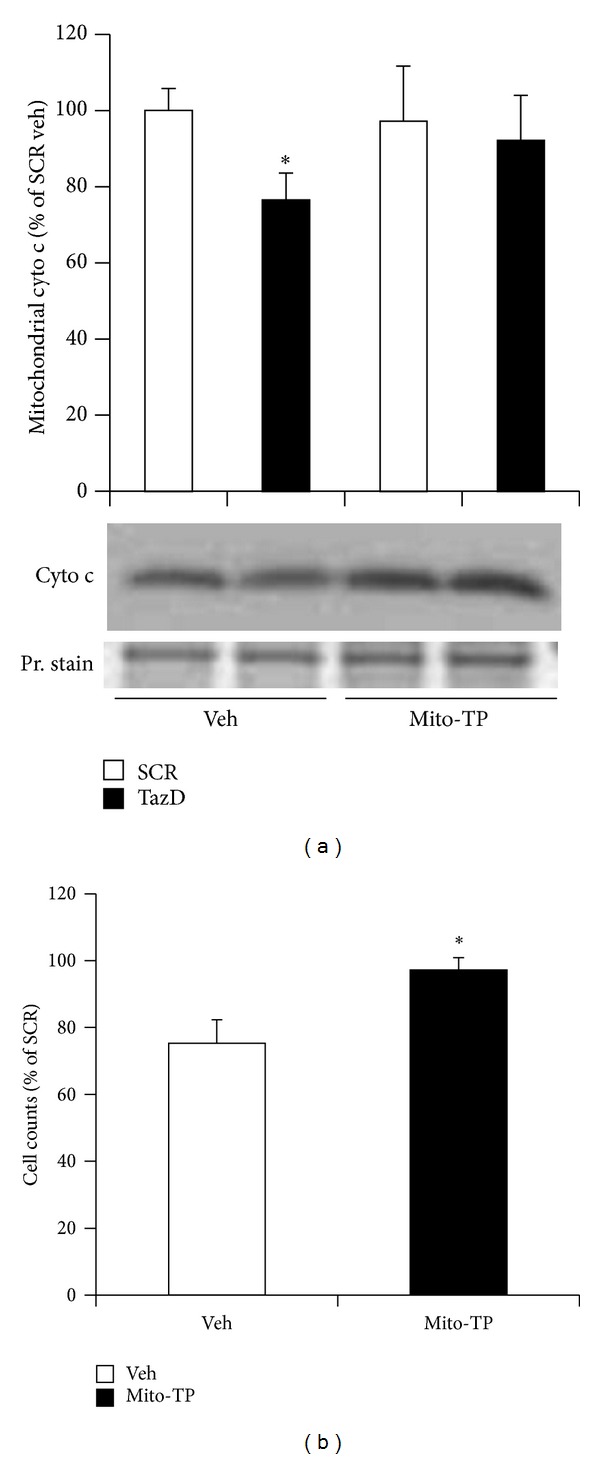
Mito-Tempo abrogated cytochrome c release and cell death induced by tafazzin knockdown. NVMs were transduced with the shRNA adenovirus for 48 h, mito-Tempo treated for the last 24 h, and tested for mitochondrial cytochrome c content and cell death. (a) Mitochondrial cytochrome c content. Data represent mean ± SE of 4 separate experiments. *P < 0.05 versus SCR treated with vehicle. (b) Cell count. Data represent mean ± SE of three separate experiments. *P < 0.05 versus vehicle.

Since cytochrome c is attached to cardiolipin in the mitochondrial intermembrane space [44], cardiolipin deficiency would be expected to cause the release of cytochrome c into the cytoplasm. As shown in Figure 7(a), cardiolipin deficiency induced by tafazzin knockdown significantly decreased mitochondrial cytochrome c content and this decrease was blocked by mito-Tempo. Cytochrome c is released to the cytoplasm* via* mitochondrial outer membrane permeabilization [44] or opening of mitochondrial permeability transition pores (PTP) [45]. Cytochrome c release from mitochondria causes cell death* via* apoptosis. Cytochrome c release from mitochondria is also a sign of PTP opening leading to cell death* via* necrosis. We next checked the effect of tafazzin knockdown on cell survival and found that tafazzin knockdown decreased cell survival to about 75% compared to cells treated with a scrambled virus. Mito-Tempo normalized this decrease to 97% (Figure 7(b)). We did not find that tafazzin knockdown increased apoptosis by Tunel staining with a kit from EMD Millipore (data not shown). Most likely this is because the decreased cellular ATP in tafazzin knockdown cells since apoptosis is an ATP- dependent process [46].

## 4. Discussion

Tafazzin plays an important role in cardiolipin remodeling in the mitochondria. Tafazzin knockdown leads to reduced cardiolipin and enhances both monolysocardiolipin and the ratio of lysocardiolipin to cardiolipin, which are consistent with previous studies involved in different model systems [9, 14, 47]. Our results from NVMs revealed that the ratio of monolysocardiolipin to cardiolipin is a more sensitive marker of tafazzin dysfunction than cardiolipin content. This notion has been proved in cultured human skin fibroblasts and bloodspots of Barth syndrome patients [47, 48]. However, the profiles of cardiolipin species are different from the adult heart where tetralinoleoyl cardiolipin (T4 18 : 2 CL) is the predominant species. T4 18 : 2 CL is one of several abundant species in NVMs. This profile of cardiolipin matched that from 1-day-old mice [49] and neonatal cardiac fibroblasts [18]. Our current cardiolipin results suggest that total cardiolipin other than specific species (e.g., T4 18 : 2 CL) is important for mitochondrial function in the early stages of the neonatal heart. This notion is also supported by a study showing that the functions of cardiolipin species are indistinguishable in yeast [50].

Cardiolipin is essential for proper function of the mitochondrial respiration chain, and dysfunction of the chain complexes leads to increased production of ROS [51]. ROS play an important role in the pathophysiology of cardiac remodeling [52]. Our previous study showed that tafazzin knockdown induced cardiomyocyte hypertrophy [17], which is most likely mediated by ROS. We have shown that tafazzin dysfunction increases ROS production in yeast and neonatal cardiac fibroblasts [22]. Our current data showing that tafazzin knockdown increased ROS production are consistent with this notion. Mitochondrial ROS have been postulated as the major resource in cardiac myocytes, since they are filled with mitochondria and ROS are byproducts of the respiration chain there. The enhanced mitochondrial ROS production directly damages mitochondrial protein, DNA, and lipids, leading to impaired mitochondrial function. As our data showed, tafazzin knockdown decreased mitochondrial ATP production and cytochrome c content. The mitochondria-targeted ROS scavenger, mito-Tempo, normalized these mitochondrial dysfunctions. Considering that tafazzin knockdown did not decrease cardiolipin dramatically though statistically significant, it is possible that tafazzin knockdown directly enhanced mitochondrial ROS production. This possibility warrants further investigation.

Our previous study showed that activation of AMPK leading to mitochondrial biogenesis involved in tafazzin knockdown induced cardiac hypertrophy in NVM since tafazzin knockdown causes mitochondrial dysfunction [17]. The increased dysfunctional mitochondria do not improve oxidative stress or cellular ATP decline. They even make the situation worsen leading to cardiac hypertrophy. Previous studies also showed that ROS activate Jak2 and thereafter STAT3 involved in cardiac hypertrophy [41, 53, 54]. Our current study showed that mito-Tempo abolished tafazzin knockdown induced AMPK activation and partial attenuated JAK2 activation implying that mitochondrial dysfunction is the major mechanism responsible for cardiac dysfunction induced by tafazzin knockdown and mitochondrial ROS triggered JAK2 signing is partially involved.

ROS mediate cardiac myocyte hypertrophy induced by mechanical stretch [55] and hypertrophic factors such as endothelin and phenylephrine [56]. Hydrogen peroxide directly induced cardiac hypertrophy [57], and antioxidants prevent hypertrophy induced by tumor necrosis factor-α and angiotensin II [58]. A recent clinical investigation showed that a supplemental antioxidant has no beneficial effects on cardiovascular diseases [59]; however, mitochondria-targeted antioxidant MitoQ10 proved effective for endothelial function improvement and cardiac hypertrophy attenuation in the stroke-prone spontaneously hypertensive rat [35]. Our current study showed that mito-Tempo blocked tafazzin knockdown induced cardiac hypertrophy suggesting that mitochondria-targeted antioxidants are promising targets for cardiac hypertrophy.

Mutation of tafazzin results in poor contractility in Barth syndrome patients [60, 61] and this phenotype has been reproduced in mice [43] and zebrafish [13]. Our data showed that tafazzin knockdown impaired contractility in cardiac myocytes and this impairment was blocked by mito-Tempo, suggesting that the enhanced mitochondrial ROS production mediates tafazzin knockdown induced contractile dysfunction.

Though cardiolipin deficiency directly results in the dissociation of cytochrome c from cardiolipin into the intermembrane space of mitochondria, extrusion of this protein still needs permeabilization of the outer mitochondrial membrane [44]. Release of mitochondrial contents including cytochrome c is a sign of PTP opening leading to necrotic cell death. Previous studies also showed that cytochrome c is released in a ROS-dependent fashion [62]. Tafazzin knockdown induced cell death but not by apoptosis. This agrees with a previous report showing that dysfunctional tafazzin does not lead to apoptosis [15, 63]. Our current study showing that mito-Tempo prevented tafazzin knockdown induced cytochrome c release and cell death demonstrates a means for mitochondrial stress induced cardiac myocyte death in cardiomyopathy.

## 5. Conclusion

In summary, our study demonstrated that tafazzin knockdown causes enhanced mitochondrial ROS production leading to mitochondrial and cardiac dysfunction, and a mitochondria-targeted antioxidant prevented the mitochondrial and cardiac dysfunction, including decreased mitochondrial ATP production, cardiac myocyte hypertrophy, contractile dysfunction, and cell death. Our results shed light on the development of mitochondria-targeted antioxidants for cardiomyopathies that resulted from tafazzin mutation and mitochondrial oxidative stress.

## Abbreviations

ROS: Reactive oxygen species
NVM: Neonatal ventricular myocyte
ACM: Adult cardiac myocyte
shRNA: Short hairpin RNA
Tempol: 4-Hydroxy-2,2,6,6-tetramethylpiperidinyloxy
Mito-Tempo: (2-(2,2,6,6-Tetramethylpiperidin-1-oxyl-4-ylamino)-2-oxoethyl) triphenylphosphonium chloride
Mn-SOD: Manganese superoxide dismutase
BDM: 2,3-Butanedione monoxime
PTP: Permeability transition pores.
